# Role of Matrix Metalloproteinase Activity in the Neurovascular Protective Effects of Angiotensin Antagonism

**DOI:** 10.1155/2014/560491

**Published:** 2014-07-24

**Authors:** Tauheed Ishrat, Anna Kozak, Ahmed Alhusban, Bindu Pillai, Maribeth H. Johnson, Azza B. El-Remessy, Adviye Ergul, Susan C. Fagan

**Affiliations:** ^1^Charlie Norwood VA Medical Center, Augusta, GA 30912, USA; ^2^Program in Clinical and Experimental Therapeutics, College of Pharmacy, University of Georgia, HM 1212, 1120 15th Street, Augusta, GA 30912, USA; ^3^Department of Biostatistics, Georgia Regents University, Augusta, GA 30912, USA; ^4^Vision Discovery Institute, Georgia Regents University, Augusta, GA 30912, USA; ^5^Department of Physiology, Georgia Regents University, Augusta, GA 30912, USA; ^6^Department of Neurology, Georgia Regents University, Augusta, GA 30912, USA

## Abstract

*Background and Purpose*. Oxidative stress and matrix metalloproteinase (MMP) activity have been identified as key mediators of early vascular damage after ischemic stroke. Somewhat surprisingly, the angiotensin II type 1 receptor (AT1) blocker, candesartan, has been shown to acutely increase MMP activity while providing neurovascular protection. We aimed to determine the contribution of MMP and nitrative stress to the effects of angiotensin blockade in experimental stroke. *Methods*. Wistar rats (*n* = 9–14/group; a total of 99) were treated in a factorial design with candesartan 1 mg/kg IV, alone or in combination with either a peroxynitrite decomposition catalyst, FeTPPs, 30 mg/kg IP or GM6001 50 mg/kg IP (MMP inhibitor). Neurological deficit, infarct, size and hemorrhagic transformation (HT) were measured after 3 h of middle cerebral artery occlusion (MCAO) and 21 h of reperfusion. MMP activity and nitrotyrosine expression were also measured. *Results*. Candesartan reduced infarct size and HT when administered alone (*P* = 0.0011) and in combination with FeTPPs (*P* = 0.0016). GM6001 did not significantly affect HT when administered alone, but the combination with candesartan caused increased HT (*P* < 0.0001) and worsened neurologic score (*P* = 0.028). *Conclusions*. Acute administration of candesartan reduces injury after stroke despite increasing MMP activity, likely by an antioxidant mechanism.

## 1. Introduction

Ischemic stroke, an obstruction of blood flow in a major cerebral vessel, remains a leading cause of adult disability and death in the United States [1]. Because of its complex pathology, a major research and clinical priority is to develop therapeutic interventions in the ischemic brain through the understanding of underlying mechanisms.

Ischemia reperfusion leads to a cascade of pathophysiological processes, resulting in further brain damage. Accumulations of free radicals, oxygen/nitrogen species (ROS/RNS), not only increase the susceptibility of brain tissue to reperfusion-induced damage but also trigger numerous molecular cascades, leading to increased blood-brain barrier (BBB) permeability, brain edema, hemorrhage and inflammation, and brain death [2, 3]. As an important component of free radicals, RNS, including peroxynitrite (ONOO–), play important roles in the process of cerebral ischemia-reperfusion injury. Ischemia reperfusion results in the production of peroxynitrite in ischemic brain, which triggers numerous molecular cascades and leads to vascular damage.* In vitro*, peroxynitrite strongly activates matrix metalloproteinases (MMPs) [4, 5]. Peroxynitrite formation on microvessels colocalizes with MMP-9 expression after cerebral ischemia [2]. MMPs, particularly MMP-9 and MMP-2, are important mediators of vascular dysfunction/remodeling after stroke [6–8]. Recent studies suggested that administration of peroxynitrite decomposition catalyst (FeTPPS) decreases infarct size, MMP activation, and neurovascular injury after cerebral ischemia [9, 10].

Blockade of the AT1 receptor has emerged as an effective strategy to prevent neuronal and vascular damage in experimental stroke [11–13] and other vascular events in patients at risk [14]. We have shown that candesartan, an AT1 receptor blocker, is neuroprotective and vasculoprotective and improves functional outcome in both permanent and transient models of stroke [11, 15–17]. These beneficial effects of candesartan are mediated through a number of different mechanisms including upregulation of eNOS expression [18], upregulation of growth factor expression [12], and amelioration of oxidative stress [19]. Moreover, we have recently demonstrated the ability of candesartan to reduce protein nitration (nitrotyrosine levels) after stroke as well as in response to peroxynitrite treatment in a cellular model of stroke, again implicating its antioxidant and antinitrative effects [20]. Unfortunately, the development of candesartan as an acute treatment strategy in stroke patients was halted when the negative results of a large clinical trial were published [21]. The authors concluded that the blood pressure lowering achieved with the drug may have explained the lack of benefit seen. The mechanism of the robust vascular protective effect demonstrated when candesartan is administered in experimental models needs to be determined so that alternative tactics to achieve the same end can be pursued.

Candesartan reduces hemorrhage formation, despite acutely increasing the activation of the matrix MMPs-2 and -9 after experimental stroke [15, 17, 22]. Even in embolic stroke treated with tissue plasminogen activator (tPA), where MMP-9 activation has been associated with increased hemorrhagic transformation (HT) and worsened outcomes in animals [23, 24] and humans [25, 26], we have shown candesartan to be acutely vascular protective [22]. Studies continue to demonstrate the variety of mechanisms and pathways by which candesartan provides neurovascular protection in stroke; however, there are still many that need to be addressed. To examine the mechanism and contribution of MMP activation and tyrosine nitration to the neurovascular protective effects of angiotensin blockade in experimental stroke (Figure 5), we set out to determine the effects of (1) candesartan alone and in combination with the peroxynitrite decomposition catalyst, FeTPPs, and (2) candesartan alone and in combination with the broad spectrum MMP inhibitor, GM6001, on infarct size, HT, and neurologic deficit after ischemic stroke.

## 2. Materials and Methods

### 2.1. Animals and Treatment Regimen

Male Wistar rats (270–300 g; Charles River Laboratories, Wilmington, MA) were used according to procedures approved by the Institutional Animal Care and Use Committee (IACUC) of the Charlie Norwood VA Medical Center.

The study was performed in two separate experiments. In both experiments, all animals received an intravenous (IV) injection of either candesartan or vehicle and an intraperitoneal (IP) injection of an additional compound (FeTPPs or GM6001) or their vehicles (saline and DMSO, resp.). Experiment I was carried out to evaluate the effects of candesartan alone and in combination with FeTPPs on HT, infarct size, edema, functional outcome, and MMP activity at 24 h after tMCAO. The groups were transient middle cerebral artery occlusion (tMCAO) + vehicle treatment group I (vehicle only; *n* = 13), tMCAO + candesartan (1 mg/kg) treatment group II (candesartan only; *n* = 14), tMCAO + FeTPPs 30 mg/kg IP treatment group III (FeTPPs only; *n* = 12), and tMCAO + candesartan + FeTPPs group IV (Cand + FeTPPs; *n* = 16). Experiment II was carried out to evaluate the effects of candesartan alone and in combination with a broad spectrum MMP inhibitor, GM6001 (Calbiochem). The groups were transient middle cerebral artery occlusion (tMCAO) + vehicle (dimethyl sulfoxide − DMSO + saline) treatments group I (vehicle only; *n* = 10), tMCAO + candesartan (1 mg/kg in saline) + DMSO treatment group II (candesartan only; *n* = 9), tMCAO + GM6001 (50 mg/kg, in DMSO, IP) treatment group III (GM6001 only; *n* = 12), and tMCAO + candesartan + GM6001 treatment group IV (Cand + GM6001; *n* = 13).

### 2.2. Temporary Middle Cerebral Artery Occlusion (tMCAO)

All animals were anesthetized with 2% isoflurane via inhalation. Cerebral ischemia was induced using the intraluminal suture middle cerebral artery occlusion (MCAO) model [27]. The right MCA was occluded with a 19–21 mm 3-0 surgical nylon filament, which was introduced from the external carotid artery lumen into the internal carotid artery to block the origin of the MCA. The animals were anesthetized for only 10 min for the surgical procedure. The suture was removed after 3 h of occlusion and the animals were returned to their cages. At reperfusion, a single dose of 1 mg/kg candesartan or saline control was given intravenously through a tail vein at a volume of 1 mL/kg. The duration of occlusion (3 h) and reperfusion (21 h) and the dose of candesartan (1 mg/kg) were chosen based on our own published data demonstrating reliable vascular injury in this model and robust neurovascular protection with candesartan [11, 16, 17].

### 2.3. Neurological Assessment

All animals underwent neurobehavioral testing before MCAO and at 24 h after MCAO. Tests that were used included Bederson score, beam walk, and paw grasp and were performed in a blinded fashion.

*Bederson Score.* Neurological function was measured before reperfusion and at 24 h (just before animals were killed) using the Bederson score [28]. Animals were assigned a score from 0–3. The animal is given a point for each of the following: forelimb flexion when suspended by tail; decreased resistance to lateral push; and contralateral circling. A score of 3 is consistent with MCAO. Only animals with a score of 3 at the time of reperfusion were included in the analysis of infarct size, hemoglobin, and neurological function.* Beam Walk*. Beam walking ability is graded 0 for a rat that readily traverses a 2.4 cm wide, 80 cm long beam to 3 for a rat unable to stay on the beam for 10 seconds.* Paw Grasp.* Bilateral forepaw grasp measures the ability to hold onto a 2 mm diameter steel rod, graded 0 for a rat with normal forepaw grasping behavior to 3 for a rat unable to grasp with the forepaws.

### 2.4. Assessment of Infarct Size, Edema, and Hemoglobin (Hb) Content

At 24 h after the onset of MCAO, the animals were anesthetized with ketamine 44 mg/kg and xylazine 13 mg/kg i.m. (cocktail), perfused with saline, and sacrificed and the brains were removed. The brain tissue was sliced into seven 2 mm thick slices in the coronal plane and stained with a 2% solution of 2,3,5-triphenyltetrazolium chloride (TTC) (Sigma Chemical Co., St. Louis, Missouri, USA) for 15–20 min. Images of the stained sections were taken. Grossly visible infarction zones were quantified using Image J analysis software (Image J, NIH) and corrected for edema. Edema was quantified as the difference in area between the hemispheres and expressed as a percentage of the contralateral hemisphere.

The ischemic and nonischemic hemispheres of the slices for the enzyme-linked immunosorbent assay (ELISA) were separated and processed. After homogenizing the slices in the core of the infarct and collecting the supernatants, enzyme-linked immunosorbent assay was performed to measure the hemoglobin content using HB ELISA kit according to the manufacturer's instructions (BioAssay systems, Hayward, CA, USA).

### 2.5. Gelatin Zymography

Substrate-specific zymography for determination of gelatinolytic activity of MMP-9 and MMP-2 was performed on brain homogenates taken 24 h after MCAO. The concentration of protein was adjusted equally in all of the tissue samples. Samples were then mixed 1 : 1 with loading buffer (80 mmol/L Tris-HCl [pH 6.8], 4% SDS, 10% glycerol, and 0.01% bromophenol blue) and left standing for 10 min at room temperature. Proteins were separated by electrophoresis in a 10% SDS-PAGE gel containing 0.1% gelatin at 125 volts constant current. Gels were then washed to remove SDS with 2.5% Triton X-100 (Sigma) for 1 h and incubated at 37°C with developing buffer (50 mmol/L Tris-HCl [pH 7.5], 10 mmol/L CaCl_2_, 0.02% NaN_3_) for 36 h. Enzymatic bands were visualized after staining for 1 h with Coomassie blue (BioRad) R-250 for 30 min and destained with three changes of methanol : acetic acid : water (50 : 10 : 40). The gel was scanned and the bands of activity were quantified using Image J analysis software (Image J, NIH).

### 2.6. Slot Blot for Nitrotyrosine

Nitrotyrosine (NT) immunoreactivity is measured as an indicator of superoxide-dependent peroxynitrite formation by slot blot analysis. Brain homogenates (20 *μ*g) prepared for immunoblotting experiments were immobilized onto a PVD membrane using a slot blot microfiltration unit. After blocking with 5% nonfat milk, membrane was incubated with an anti-nitrotyrosine antibody from Calbiochem and visualized with Pierce Super Signal Kit. The intensity of bands was analyzed by GelPro Software.

### 2.7. Statistical Analysis

Data are presented as mean ± SE. The normality and homogeneity of variance assumptions of ANOVA were assessed and a rank transformation was used if needed. Data were analyzed using a 2-candesartan (vehicle versus yes) × 2 FeTPPs or GM6001 (vehicle versus yes) ANOVA with interaction using SAS 9.3 (SAS Institute, Inc., Cary, NC). Statistical significance was determined at alpha = 0.05.

## 3. Results 

### 3.1. Candesartan Mimics the Peroxynitrite Scavenger in Reducing Nitrotyrosine after tMCAO

**Figure 1:**
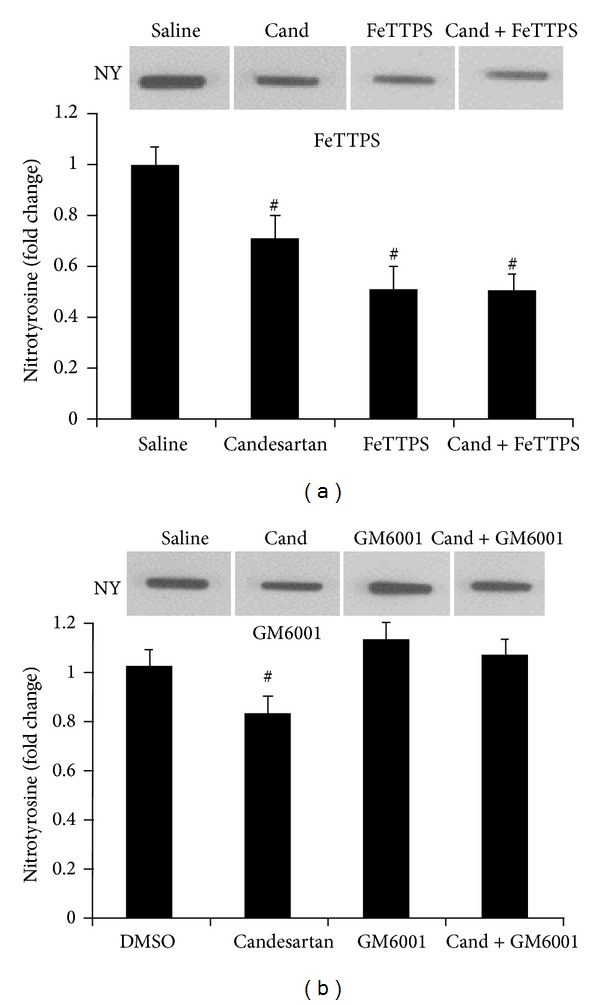
Effects of candesartan alone and in combination with FeTPPS and GM6001 on nitrotyrosine (NT) at 24 h after MCAO. Candesartan, FeTTPS, and their combination demonstrated reduced NT compared to the saline treated group (^#^ *P* < 0.05). Candesartan alone reduced NT compared to DMSO and GM6001 alone, but the candesartan combination with GM6001 blunted the effect of candesartan. Values are mean ± SEM (^#^ *P* < 0.05).

Nitrotyrosine (NT) is a product of free radical oxidation of nitric oxide damage under a variety of disease conditions including stroke [29]. FeTTPS administration significantly (*P* < 0.05) reduced NT at 24 h after the onset of MCAO (Figure 4). Candesartan alone also significantly (*P* < 0.05) reduced the NT but the combination was not significantly different from FeTTPs alone. In the GM6001 experiment, candesartan alone significantly (*P* < 0.05) reduced NT compared to all groups but not when administered with GM6001 (Figure 1).

### 3.2. GM6001 Blunts the Candesartan-Induced MMP Activation after MCAO

**Figure 2:**
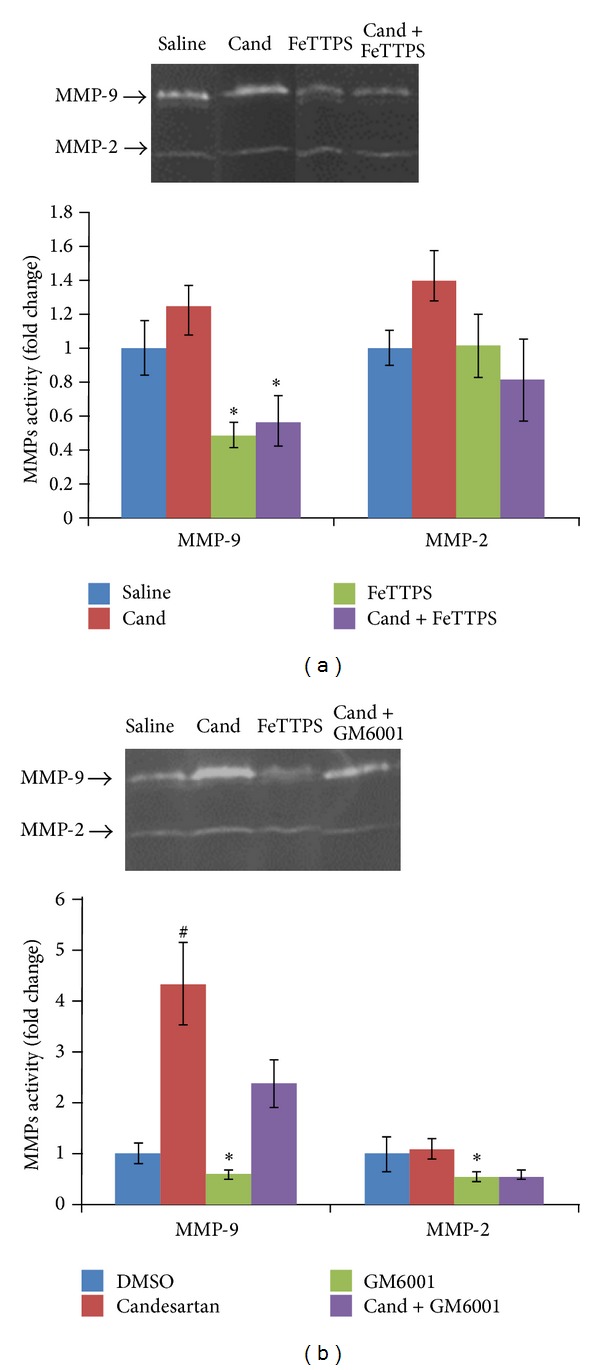
Effects of candesartan alone and in combination with FeTPPS and GM6001 on MMP activity at 24 h after MCAO. MMP-9 was significantly inhibited by FeTTPS alone and in combination with candesartan. Candesartan alone significantly increased MMP-9 when administered with DMSO, but GM6001 significantly reduced MMP activity. Values are mean ± SEM (^#^ *P* < 0.05, *P < 0.05).

Consistent with our previous studies [16, 17], gelatin zymography after MCAO showed upregulated MMP-9 and MMP-2 in the vehicle-treated animals compared with sham-operated controls. As we have previously reported [17], a single dose of candesartan at reperfusion significantly increased MMP-9 activity (*P* < 0.0001) in the ischemic hemisphere. As expected, GM6001 administration significantly reduced MMP-2 and -9 activity (*P* < 0.05) at 24 h after the onset of MCAO (Figure 2). The effect of the combination was intermediate, revealing the ability of GM6001 to blunt the upregulation of MMP-9 activity due to candesartan.

In addition, as we and others have reported [9, 30], peroxynitrite decomposition catalysts prevent MMP activation after cerebral ischemia. We again found that FeTPPs reduced MMP-2 and -9 activation, both alone and in combination with candesartan (Figure 2).

### 3.3. Candesartan Alone and in Combination with FeTPPS Improves Functional Outcome after tMCAO

**Figure 3:**
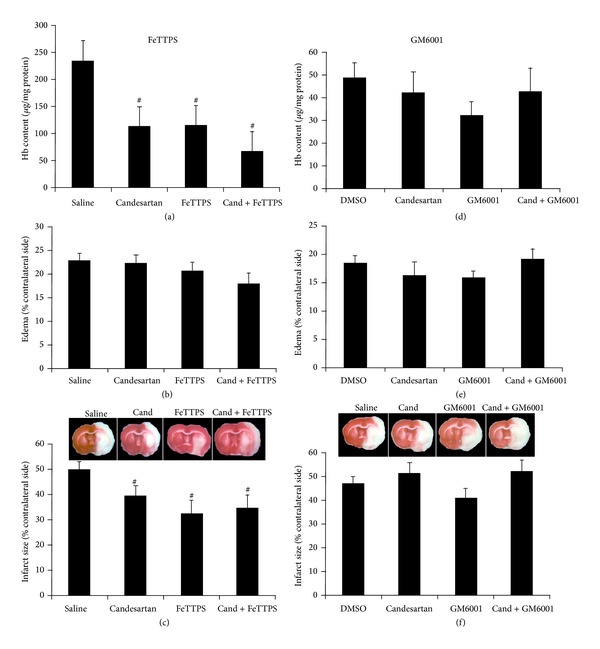
Effects of candesartan alone and in combination with FeTPPS and GM6001 on infarct size (a, d), edema (b, e), and hemoglobin content (c, f) at 24 h after MCAO. Infarct size and Hb content were significantly reduced in all three treatment groups in FeTTPS compared to the saline treated group (^#^ *P* < 0.05). Candesartan alone and in combination with GM6001 did not reduce these outcomes when administered with DMSO, but GM6001 alone resulted in a significantly reduced infarct size. Values are mean ± SEM (^#^ *P* < 0.05).

**Figure 4:**
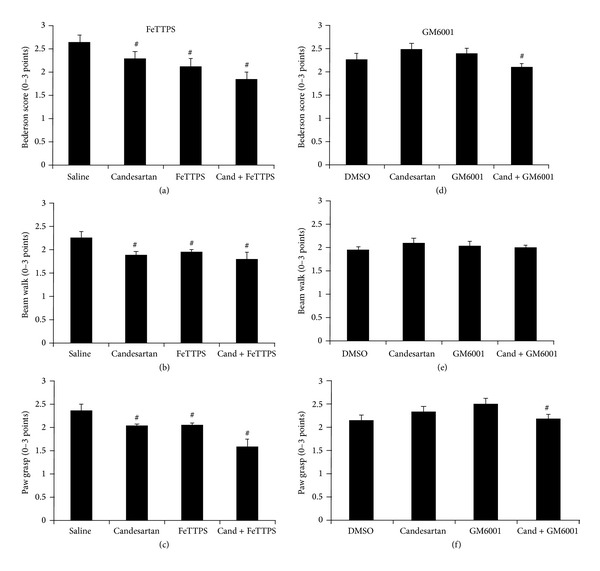
Effects of candesartan alone and in combination with FeTPPS and GM6001 on neurobehavioral tests: Bederson scores (a, d), beam walk (c, e), and paw grasp (c, f) at 24 h after MCAO. All three treatment groups in FeTTPS demonstrated improved behavior compared to the saline treated group (^#^ *P* < 0.05). Candesartan did not affect neurobehavioral outcomes when administered with DMSO, but the combination of candesartan with GM6001 resulted in significantly improved outcomes in Bederson score and paw grasp. Values are mean ± SEM (^#^ *P* < 0.05).

**Figure 5:**
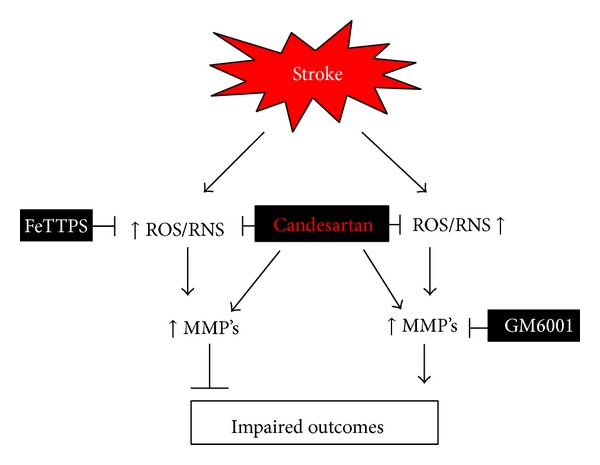
Schematic of experimental hypothesis for the possible mechanisms of candesartan-induced acute protection. ROS: reactive oxygen species, RNS: reactive nitrogen species, MMP: matrix metalloproteinases, FeTPPs: a peroxynitrite decomposition catalyst, 5,10,15,20-tetrakis (4-sulfonatophenyl)porphyrin to iron III chloride, and GM6001: a broad spectrum MMP Inhibitor.

Both candesartan and FeTPPs significantly reduced bleeding (HT) at 24 h (*P* < 0.01), and FeTPPs alone and in combination reduced infarct size (*P* = 0.012). All three treatment groups demonstrated improved behavior compared to the saline treated group (*P* < 0.05). There was no significant interaction detected between candesartan and FeTPPs (Figure 4). Candesartan, however, when combined with GM6001 or its vehicle, DMSO, resulted in significantly higher infarct size (*P* = 0.041) and a trend toward increased bleeding (HT) (Figure 3). This was manifested in a significantly worse functional outcome in terms of neurologic score (*P* = 0.028) and paw grasp (*P* = 0.022) in the candesartan-treated groups (Figure 3). There were no significant differences in edema formation in any of the treatment groups.

## 4. Discussion

The neuroprotective effects of the angiotensin receptor antagonists are well documented in experimental cerebral ischemia [13], but their robust vascular protective effects [11, 17, 31] appear to be incompatible with the demonstrated ability to increase MMP-2 and MMP-9 activity [17]. The experiments reported here represent attempts to determine the mechanism of the early vascular protective effects of candesartan so that other interventions can be developed that do not have the attendant blood pressure lowering properties.

Reperfusion after ischemia increases levels of reactive oxygen species, including superoxide radical [32] and nitric oxide (NO) [33]. Oxidative radicals trigger activation of MMPs after ischemic stroke [34]. Superoxide reacts with NO to form peroxynitrite. Oxygen-glucose deprivation or reperfusion after cerebral ischemia increases peroxynitrite in cerebral endothelial cells and neurons [35, 36]. Peroxynitrite is more permeable through the lipid membrane than superoxide [37] and is more toxic [38].* In vitro*, peroxynitrite strongly activates MMPs including MMP-9 and MMP-2 [4, 5]. After ischemia reperfusion, peroxynitrite formation on microvessels leads to MMP-9 activation and neurovascular damage (hemorrhage and edema) by disrupting microvascular integrity [2]. Moreover, administration of FeTPPS decreases MMP activation and neurovascular injury through decompositing peroxynitrite levels after cerebral ischemia [9]. In the present study, we test whether administration of a peroxynitrite decomposition catalyst alone and in combination with candesartan after ischemic stroke decreases neurovascular injury. We observed that candesartan mimics the peroxynitrite scavenger in reducing nitrotyrosine.

The utility of broad spectrum MMP inhibitors, like GM6001, for experimental work and therapeutic use has been severely limited by their water insolubility. The need to dissolve the agents in DMSO, as we have done in the experiments reported here, introduced unintended consequences of the vehicle itself. DMSO is a nonspecific free radical scavenger that is known to be neuroprotective [39], so any investigation involving its use as a solvent requires a DSMO control group as we have done. The impact of DSMO on the postischemic cerebral vasculature is less clear. After 1.5 h of MCAO and 48 h of reperfusion in Wistar rats, DMSO reduced blood-brain barrier (BBB) disruption as measured by MRI [40] but in Sprague-Dawley rats subjected to 2 h of MCAO and 2 h of reperfusion, DMSO significantly reduced BBB integrity [41]. In our experiments, DMSO alone did not have a measurable protective effect on either infarct size or HT. However, DMSO when administered IP at the same time as candesartan IV resulted in an increase in infarct size and a worsened neurologic outcome. If we had studied only the combination of candesartan with GM6001, compared to candesartan alone and saline, we would have concluded that the vascular protective effects of candesartan were dependent on MMP activation. Use of the DMSO control, however, makes it clear that this is not the case. The negative interaction between GM6001 and candesartan is entirely explained by the organic solvent where it is dissolved in. The early neurovascular protective effects of candesartan, administered at reperfusion, occur despite, not because of, the increase in MMP activation.

A more likely candidate mechanism for the acute protective effects of the ARB is their known antioxidant effects [42, 43]. In this investigation, the peroxynitrite decomposition catalyst, FeTPPs, provided potent neurovascular protection when administered at reperfusion, and this was consistent with other reports [9, 10]. The addition of FeTPPs, although effective alone, did not add to the benefit seen with candesartan.

In this investigation, we have shown that the neurovascular protective effects of candesartan were matched and maintained when compared to FeTPPs alone and their combination, respectively. This occurred despite the ability of FeTPPs to reduce MMP activation. However, when candesartan was combined with GM6001 or its vehicle, DMSO, the beneficial effects of candesartan were lost, manifesting as an increase in infarct size and HT and a worsened neurologic outcome. This worsening occurred despite an effective reduction in MMP-9 activity in the GM6001-treated groups.

We have shown that the proangiogenic and trophic effects of candesartan in the brain vasculature are mediated through AT2 stimulation [44]. We subsequently proposed that the enhancement of long-term recovery after candesartan [16] may be achieved with a direct AT2 agonist, avoiding the BP lowering effects of AT1 receptor blockade. An important limitation to the development of AT2 agonism as a therapeutic strategy for acute ischemic stroke may be the lack of acute antioxidant properties [43], although other acute benefits may be achieved and are under investigation. This study focused on the acute-stage treatment of ischemic injury and did not include long-term follow-up. Further investigations are ongoing to determine the mechanisms responsible for the long-term benefits of candesartan and other modulators of angiotensin signaling after stroke.

In summary, the acute neurovascular protective effects of candesartan occur despite increases in MMP activity and are likely to be at least partially explained by an acute antioxidant effect. The increase in MMP activity seen after candesartan administration may be necessary to promote long-term recovery after the acute period.
